# Clinician Distress in the Care of Seriously Ill Hospitalized Patients: A Mixed Methods Integration of Qualitative Findings and a Quantitative Typology

**DOI:** 10.21203/rs.3.rs-10272024/v1

**Published:** 2026-08-07

**Authors:** Anessa Foxwell, Salimah Meghani, Janet Deatrick, Katherine Courtright, Liming Huang, Joseph Rhodes, Karen Hirschman, Connie Ulrich

**Affiliations:** University of Pennsylvania

**Keywords:** clinician distress, emotional well-being, serious illness, palliative care, mixed methods

## Abstract

**Background:**

Caring for seriously ill hospital patients can cause psychological, emotional, moral, ethical, spiritual or existential distress. Yet little is known about the manifestation and experience of clinician distress, the triggers for distress, and how clinicians seek support in real-time. In preliminary analyses, cluster analysis of 142 encounters yielded four distinct clusters which formed a quantitative distress typology: low, moderate, variable, and high which needed further validation and development.

**Research purpose:**

This study aims to further develop the clinician distress typology by using quantitative and qualitative data to validate and describe the four clusters.

**Research design & methods:**

A mixed methods (QUANàqual) explanatory sequential design was conducted. Qualitative data augmented the identification of distress typologies by providing context and a richer understanding of the clinicians’ experience of distress.

**Participants and research context:**

Hospital clinicians (n=25) were interviewed, and qualitative themes examined the experience of distress by typology. Mixed analysis confirmed typologies with inductive themes and mean distress thermometer score (DT1) as well as clinician emotional experience mean (Gallup) score.

**Findings & Conclusion:**

Mixed analysis supported the clusters in the typology and expanded understanding of the depth and breadth of emotions experienced by clinicians caring for hospitalized seriously ill patients: (i) low distress typology theme of *“equanimity…but not like a robot”*(DT1=0.73; Gallup=0.53); (ii) moderate distress typology theme of *“tightrope”*(DT1=2.98; Gallup=2.05), (iii) variable distress typology *“comfortable in the chaos”* (DT1=3.25; Gallup=2.19), and (iv) high distress typology inductive code of *“weight/waiting”* (DT1=5.79; Gallup=3.55). Clinicians were less distressed if they felt supported in a positive work environment. This mixed methods study advances the understanding of clinician psychological distress and has vital implications for healthcare systems developing workplace resources for support.

## Introduction

Caring for seriously ill patients can cause clinicians to experience distress.^1-5^ Clinician emotional reactions could occur during or after a patient-clinician interaction and may lead to distress—whether psychological, emotional, moral, ethical, spiritual or existential. However, fostering clinician recognition and attention to emotions in serious illness care is a relatively new recommendation.^6,7^ And little is known about the manifestation and experience of clinician distress and how it may impact care.

Most research on distress focuses on the concept of moral distress in response to an ethical dilemma. Clinician distress—particularly stemming from a psychological or emotional reaction—has been described by clinicians themselves in editorial pieces.^8-16^ These narratives necessitate further inquiry as they describe clinician distress that impacts the therapeutic relationship and empathic communication. And there is no standardized way to support those experiencing clinician distress, despite national organizations such as the National Academy of Medicine and the American Nurses Association, advocating for both individual and systemic interventions for clinician well-being.^17,18^

A mixed methods approach is well-suited to look more closely at these patterns of clinician distress in serious illness care as the problems are complex requiring inquiry into both the how and the why.^19^ Quantitative work serving as the first arm of this study begins to establish a pattern and sequelae of clinician distress.^2^ Through the collection of both quantitative and qualitative data, triangulation of findings strengthen the understanding of the phenomenon of clinician distress.^20-22^

## Study Design

A mixed methods (QUAN◊qual) explanatory sequential study was completed. The quantitative design and findings are described elsewhere.^2^ Four clinical distress trajectories or typologies were identified via cluster analysis.^2^ Then, the experience of distress, sources and supports were explored through in-depth semi-structured interviews with clinicians from each typology: (i) low distress trajectory, (ii) moderate distress trajectory, (iii) variable distress trajectory, and (iv) high distress trajectory (see Appendix I: Semi-Structured Interview Guide). Quantitative data was collected with mobile ecologic momentary assessment (mEMA) to inform the qualitative interviews with clinicians who offered narratives of their express of distress. Quantitative (typologies) and qualitative (clinician distress themes analyzed by qualitative descriptive methods) data were integrated using thematic matrix analysis to further elucidate how qualitative themes are distributed across the levels of distress typologies. This study was approved the University of Pennsylvania Institutional Review Board (#852340).

### Sample & Data Collection

#### Recruitment.

Clinicians who are caring for seriously ill hospitalized patients were identified via a clinical prediction morality risk model.^23,24^ Complete recruitment details were previously published.^2^ For qualitative recruitment, a question was built-in to the final mEMA survey asking if the participant would be interested in a follow-up interview. A total of 36 participants opted-in and were contacted via email or secure electronic health record (EHR) chat. Twenty-five participants completed interviews between July 18, 2023, and October 28, 2023. A Distress Protocol (see Appendix II: Distress Protocol) was developed based on previous work in case of emotional distress during interviews.^25,26^

#### Measures.

Measures will be briefly described here; for more detail, see previously published work.^2^ The Distress Thermometer (DT) is an 11-point visual analog scale (VAS) used to screen for distress across various populations.^27-33^ The Gallup negative experience index^34^ measured clinician emotional experience index through a self-report tool. The Gallup negative experience index includes seven questions asking one-day recall of psychological and emotional symptoms (i.e., insomnia, pain, worry, sadness, anger, stress, fatigue), answered with yes/no. Multiple symptoms or “yes” answers indicated high level of symptom acutely. Clinicians were finally asked about system factors, namely job strain, workload, perception of climate, and perceived support; each on an 11-point scale of poor (0) to outstanding (10). These were asked one-time after all mEMA data was collected. Patient demographics are not reported here.

### Data Analysis

#### Qualitative Data Analysis.

All participant transcripts were cleaned and de-identified then stored in NVivo software^35^ on a secure server at the [blinded university]. A deductive-inductive approached was used for qualitative descriptive analysis in an iterative approach.^36-38^ First, for each transcript a case summary was created informed by quantitative and qualitative data. Then, a codebook was created informed by the interview guide;^35^ inductive codes were identified during first level coding and developed into themes during second level coding.^39,40^ Transcripts were categorized by distress trajectory, as identified by cluster analysis in original analysis. Trustworthiness was established through credibility, confirmability, and triangulating findings with qualitative experts (CU, SM, JD).^36,41,42^ To maintain validity, participant quotes were minimally transformed.^20^

#### Mixed Data Analysis.

Qualitative data was analyzed as above. The primary measure for the original quantitative study was “clinician distress” which was longitudinal as recorded for each clinician through six mEMAs.^2^ The goal was to cluster clinicians based upon distress typologies, using a novel approach, “kmlShape,” specifically proposed to cluster longitudinal data.^43,44^ This approach resulted in clusters based on trajectories with 4-resulting typologies. Integrated data analysis occurred by mixing clinician quantitative and qualitative data. To gain insight on the complex phenomenon of clinician distress typologies, qualitatively-identified themes were triangulated with typologies. Quantitative and qualitative data was merged with a triangulation approach for comprehensiveness analysis of the phenomenon.^45,46^ Informational matrixes were developed to visually display data and presented in findings.^22,45,47,48^ The goal of integration was to consider how merged results provide a better understanding of clinician distress and impact on patient care. Integration yielded mixed method meta-inferences interpreted as confirmed, discordant, expanded and/or illustrated within joint displays.^20,45-48^

## Findings

The mixed analysis combines the quantitative findings with the qualitative findings for mixed interpretation. Clinician demographics from the quantitative arm are reported elsewhere.^2^ Notably the numbers of clinician responses (n=142) in each distress trajectory was between 28 and 47 observations: low distress trajectory (n=33), moderate distress trajectory (n=47), variable distress trajectory (n=28), and high distress trajectory (n=34). Then qualitative interviews were conducted with participants from each of these clusters (total of 25 participants) with a distribution of low distress typology (n=8), moderate distress typology (n=8), variable distress typology (n=5) and high distress typology (n=4). Clinician demographics of the qualitative arm are listed in **Table 1**.

### Main Findings.

A joint display by typology with an inductive code describing respective typologies paired with the number of quantitative observations from each typology, the mean of the first reported distress thermometer (DT; 0-10 scale) and the mean of the clinician emotional experience (as measured by the Gallup Negative Experience Index, 0-7 composite score) is presented in **Table 2**. The inductive theme for the low distress typology is *“equanimity…but not like a robot”* which is confirmed in the typology average low distress thermometer at time point one (DT1; mean 0.73) and low Gallup composite score (mean 0.53). For the moderate distress typology, the inductive theme *“tightrope”* where they try not to falter along the emotional balance tightrope is confirmed by the moderate DT1 (mean 2.98) and Gallup composite (mean 2.05). The inductive theme for the variable distress typology *“comfortable in the chaos”* is confirmed in the exemplar quotes presented and the slightly elevated DT1 (mean 3.25) and composite Gallup score (mean 2.19) between the moderate and high typologies. Finally, the high distress typology inductive code is *“weight/waiting,”* where clinicians both carry emotional weight and feel like they are waiting on others to complete their jobs at times. Their initial distress level is highest (DT1, mean 5.79) and they have the highest symptom burden (Gallup, mean 3.55) experiencing three to four symptoms most of the day yesterday, thus, confirming the typology. Notably, in the qualitative interviews this group was all APPs, therefore they felt constrained and distressed by *“waiting”* on others to carry out clinical decisions at times (i.e., waiting for an oncologist to decide on next course of treatment).

The experience of distress is further illustrated through case summaries (**Table 3**). The concepts from the interview guide are listed in the top left of the table with notes from the specific transcript (qualitative findings). On the top right, the individual distress trajectory is mapped with DT values over the two days that the participant was enrolled (quantitative findings). Then, important quotes of the distress experience are captured. During the qualitative interviews, the individual chart was shared with the participant allowing rich discussion of longitudinal scores and the lived experience while enrolled. The exemplar quotes included in **Table 3** include explanations of the individual graph. Finally, the mixed methods interpretation which illustrates the distress experience is found at the bottom of **Table 3**.

### Sources of Support.

Another concept that was explored both quantitatively and qualitatively was perception of support. In a joint display (**Table 4**) these sources of support are presented by typology including types of support collated from interviews and a mixed interpretation with exemplar quotes. In the quantitative surveys, participants were asked to rate their perceptions of support, work environment, and work climate; all three were asked on a visual analog scale from poor (0) to outstanding (10). Means from each typology are listed in **Table 4**; notably, the higher the distress typology level the lower the perceptions of all support, except for the variable and high typologies which have comparable means for these measures. Across typologies, the types of support included professional (colleagues and structured supports including debriefings or therapists) and personal (partner) supports.

### Mixed Distress Experience.

**Figure 1:**
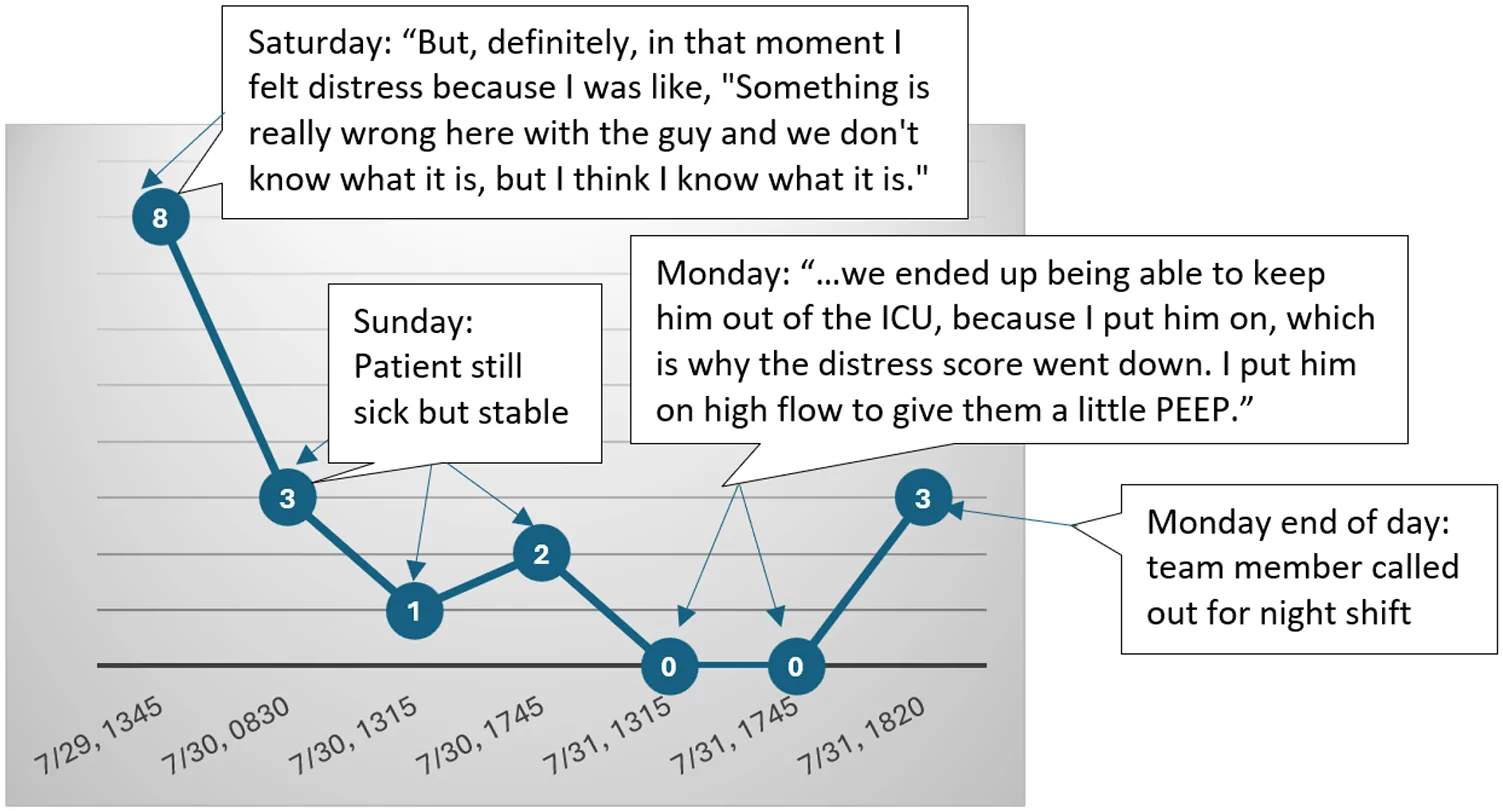
Joint Display: Mixed Distress Experience

Clinicians experience of distress is influenced by many factors—patients, workload, other responsibilities, colleagues. This “mixed” distress experience emerged from qualitative interviews. One way to illustrate this finding is through a joint display. Figure 1 includes the distress trajectory for one APP participant enrolled in the study in the variable distress typology. DT ranges from 0-8 (on the 11-point scale) for this participant over the course of a weekend. Quotes explaining the trajectory are imposed on the graph. This participant first describes an unstable patient who is contributing to their distress (DT = 8). They call this patient “*peri-ICU*” because while the patient was on the medical-surgical unit they were tenuous and on the verge of transfer to the ICU. On day 2 (Sunday), the participant describes the “*peri-ICU*” patient:

“Yeah. So then, at that point, it was probably more of just resignation to what was happening. Like, ‘Okay. He's temporized. We're probably not fixing anything this weekend,’ because it was one of those things where we were thinking, like, ‘Okay. Because if we're going to scan him and give him contrast, that's a bigger conversation that's going to have to happen with us, his oncologist, the patient, and the family, all of that. And realistically, that's not happening on Sunday. That's a Monday conversation.’”

Clinician distress decreased when the patient was stable, there was a contingency plan if they declined, and there was a plan for a family meeting on Monday. Then on Monday, the clinician distress remained low, however it spiked again due to a logistic and team stressor, rather than patient care.

### Clinician Emotional Experience.

As previously discussed, the clinicians’ emotional experience was measured by the Gallup Negative Experience Index (“Gallup”) which included seven emotional and physical symptoms. In each qualitative interview, clinicians described unique physical and/or emotional symptoms they experienced when distressed. A joint display (**Table 5**) depicts where on the body these symptoms were experienced as described by exemplar quotes, as well as the prevalence (%) and association (c^2^) with 4-cluster typologies from lowest distress to highest distress typology.

While not all symptoms proved statistically significant, all symptoms were present in qualitative interviews. The composite Gallup score is denoted by “*All emotions*” as this score was statistically significant. Another emotional symptom that was not captured by the Gallup survey but was brought up in interviews is “fun.” One participant states:

“The other thing I think that is very important to me at work, especially because we work so much, is having fun and I think seeing humor in things, and obviously, never at the expense of anyone. But sometimes fun things happen. And I think being able to just laugh at the silly things that are happening, that's sort of I think...I guess levity within the darkness is an important emotion to me.”

[429, mod]

In terms of physical symptoms, sometimes the symptom was difficult to describe as this participant notes:

“I definitely get a feeling that I don't even know how to put into words, but like almost like a shock wave kind of thing that I can feel. Or I get like abdominal pain, like sometimes I'm watching something on TV, or like reading a story about someone like it can make me have like, like, really transient, like, abdominal pain.”

[242, mod]

The clinician emotional experience was clearly salient as reported in the quantitative findings, the qualitative interviews provided rich descriptions of these symptoms and elucidated the varied experience and manifestations of these symptoms of distress.

## Discussion

Mixing of the data allowed for a deeper interpretation of the unique typologies where distress scores, emotional experience (Gallup) scores and inductive codes could be compared more closely. These findings confirmed that lower distress typologies strive to toe the line of mental calmness (“*tightrope*” and “*equanimity…but not like a robot*”) and have lower distress scores as well as lower experiences of emotions. On the other hand, the higher distress groups acknowledge the “*chaos*” of the day and are “*waiting*” for disaster or a “fire” they would have to attend to; these two groups have higher distress scores and higher emotional burden. Previous studies have looked distress in clinicians, primarily in terms of their experience of moral distress. These studies are typically cross-sectional and often distressful situations deal with an ethical issue surrounding end-of-life care.^4,49-54^

In qualitative interviews, end-of-life issues were rarely discussed as distressing. Rather, the common triggers are discharge pressure, interruptions (from phone calls or EHR messages), tasks (*“getting pulled in a million directions” [461]*) and being short-staffed or turnover. For the variable and high distress typologies, clinicians mentioned heavy caseloads and very sick patients or intense families. The low distress typology was more distressed by *“high touch” [267]* patients or families that took them away from other patients and responsibilities. Psychological distress in healthcare workers has been primarily described in times of crisis, such as pandemics where healthcare workers have an increase of anxiety, depression, and risk of post-traumatic stress disorder.^55-63^ Interestingly, the low distress typology group discussed more protectors of distress—working with a good team, having working experience, having other roles, and goal concordant care—but those protectors were mentioned less and less as distress typology increased.

Each clinician interviewed experienced one or more physical or emotional symptom. Emotions spanned the typologies. Previous research has focused on the activation of emotions with a focus on negative emotions that clinicians experience in situations of moral distress.^1,64-66^ For instance, research previously demonstrated that medical residents had drop in empathy, increase in distress, and spent less time with patients by the end of their oncology rotations; so triggered by a particularly distressing specialty rotation.^67,68^ One study of attending emergency medicine physicians found that physicians commonly had emotional symptoms (i.e. sadness and disappointment) and physical symptoms (i.e. insomnia and fatigue) triggered after a patient death.^69^

Another finding of this current study shows that perception of support was inversely related to the level of distress. Those with higher distress had poorer perceptions of institutional support and environment. Qualitative interviews confirmed that all participants sought support in an ad-hoc manner. This is consistent with previous research where physicians primarily sought support from colleagues or talking with friends or family.^69^ Interdisciplinary clinicians with a strong daily spiritual practice are less likely to experience burnout.^70^ Research shows that strong intra- and inter-disciplinary support can be beneficial.^71,72^ With recent acknowledge of structural issues impact burnout,^17,18,73^ there is an opportunity for healthcare institutions to create structured support. Some institutions have existing supports such as moral distress consult teams, “code lavender,” ethics support, and employee assistant programs.^74-78^ Structured debriefing may increase clinician empowerment and decrease distress.^79-82^ Findings point to further opportunities for structured support based on unique distress typology.

### Implications

Institutions can be charged with improving the work environment and climate. In fact, a better work environment for clinicians is associated with improved patient satisfaction.^83^ In this study the variable and high distress typologies feel the least supported and have the poorest perception of their work environment. Institutions can partner with clinician to make small changes to improve their environment. As one clinician said:

“Like most workplaces, you can find that with that comes more work that needs to be done. So you get more work piled on within the day but your day's not longer again, and it gets more stressful. And at some point it's like, where's the breaking point? How much more can we do? Or is this even sustainable?”

[150, variable]

Leadership can work directly with clinicians to examine workload and offer recognition for a job well done and reassess role sustainability. Multiple clinicians who had other responsibilities within their job description explained that the variety of roles was reenergizing for their inpatient rotations. Health systems have a chance to accomplish varied schedule for both physicians and APPs.

Research implications are numerous. Future research should explore potential interventions that could be employed by clinicians earlier to recognize emotional reactions. There have been promising interventions with individual and team-based tools such as psychological first aid,^84-87^ and meaning-centered psychotherapy for healthcare providers.^88-90^ Additionally, future quantitative work should include variables exploring the level of experience and levels of training given the key findings of this research. For further exploration of emotional distress, the Gallup tool could be useful tool for near-real time recall of symptom severity. Future studies could explore the level of patient distress to explore whether patient emotional distress affects level of clinician distress. Additional variables of interest could include patient level of trust,^91,92^ measuring burnout,^93,94^ and clinician tolerance of uncertainty.^95,96^ Uncertainty for the future is a key aspect of anxiety or worry, therefore exploration of tolerance of uncertainty could provide more context to levels of worry reported in this sample.

From a policy perspective, the findings from this work could be instrumental in reevaluating APP workflow, staffing, daily tasks, and discharge process as identified in qualitative interviews. For instance, improving APP staffing ratios could help mitigate some of the distress experience. Previous work establishing nursing staff ratios has proven that higher levels of moral distress and burnout when nurses are overworked.^97-99^ Professional organizations should lobby for policy to address clinician distress and prevent downstream effects like burnout and turnover. The National Academy of Medicine^17^ recognizes that clinician well-being is a priority; NAM and other organizations need to support policy to address clinician distress including psychological care and resources.

While this study focused on prescribing providers, we know that distress also impacts registered nurses, social workers, chaplains and other healthcare workers. Nurses were not included in this study as the “dose of care” is not equal between bedside nurses and prescribing providers. Yet given the prevalence of moral distress and burnout amongst nurses, it is pertinent to explore the in-the-moment distress of bedside nurses. There are important implications for nursing and medical education to teach mitigation strategies for distress in training and to acknowledge and normalize emotional reactions in healthcare workers caring for seriously ill people. Furthermore, for this study it is evident that workplace emotional distress intersects with moral distress; it is critical to further explore this overlap—commonalities and distinctions in order to guide future interventions for both general clinician distress and moral distress.

### Strength & Limitations

We recognize that this study has both important strengths and limitations. First, in this sequential mixed methods study, the qualitative data confirmed the quantitative data. The qualitative interviews were rich with a diverse group of clinicians in years of experience, credentials, and across identified typologies. Further, the mixed interpretation elevates the findings to provide illustrative understanding of the experience of the unique distress typologies. Lastly, many of the interview participants reported the interview itself as a therapeutic outlet to discuss and debrief on human aspects of clinical care.

In terms of limitations, this study was conducted at one health system and therefore is not generalizable nationally or internationally. While each typology was represented in the qualitative arm, the variable and high distress typologies had less participants than the low and moderate groups. Finally, this mixed methods study had dependent arms where the qualitative arm dependent on the success of the quantitative data collection; the quantitative arm was adequately powered, although did have its own attrition limitations given the competing commitments for busy clinicians.

## Conclusion

Hospital clinicians experience distress in variable ways throughout a workday. Qualitative data provided context to the narrative of clinician distress, including important triggers, the emotional experience and sources of support. Higher distress was inversely related to perception of support with the healthcare system; here lies an opportunity to improve institutional environments to mitigate clinician distress and burnout. We know that clinicians caring for seriously ill hospitalized patients experience variable levels of distress; this study offers a path forward to titrating interventions for clinician distress based on typology and clinician-identified needs.

## Supplementary Material

This is a list of supplementary files associated with this preprint. Click to download.

- Table1to5.docx
- APPENDIXI.docx

## References

[1] Foxwell AM, Meghani H, Ulrich SM. Clinician distress in seriously ill patient care: A dimensional analysis. Nurs Ethics. 2021;096973302110032. 10.1177/09697330211003259. PMC8866161 34427135

[2] [Blinded].

[3] Luitingh TL, Williams M, Vemuri S. Moral-Uncertainty Distress in Palliative Care: A Reflection on its Impact on Clinical Practice. J Pain Symptom Manage Oct. 2024;68(4):e333–7. 10.1016/j.jpainsymman.2024.07.032. 39097245

[4] Wolf AT, White KR, Epstein EG, Enfield KB. Palliative Care and Moral Distress: An Institutional Survey of Critical Care Nurses. Crit Care Nurse Oct. 2019;39(5):38–49. 10.4037/ccn2019645. 31575593

[5] Maffoni M, Argentero P, Giorgi I, Hynes J, Giardini A. Healthcare professionals' moral distress in adult palliative care: a systematic review. British Med J Supportive & Palliat Care Sep. 2019;9(3):245–54. 10.1136/bmjspcare-2018-001674. 30636204

[6] Rosenberg LB, Brenner KO, Jackson VA, The Meaning of Together: Exploring Transference and Countertransference in Palliative Care Settings. J Palliat Med Nov. 2021;24(11):1598–602. 10.1089/jpm.2021.0240. 34491110

[7] Shalev D, Traeger LN, Doyle K, Turning the Lens Inward: The Psychological Elements of Clinician Well Being. J Palliat Med Mar. 2022;25(3):349–54. 10.1089/jpm.2021.0548. 35085468 PMC10331146

[8] Baruch J. When Sensitivity Is a Liability. N Engl J Med. 2020;382(8):694–5. 10.1056/nejmp1911401. 32074417

[9] Loren AW. Harder to Treat Than Leukemia - Opioid Use Disorder in Survivors of Cancer. N Engl J Med Dec. 2018;27(26):2485–7. 10.1056/NEJMp1812850. 30586517

[10] Thomas Jd. How Far Along Are You? Ann Intern Med. 2007;147(10):738. 10.7326/0003-4819-147-10-200711200-00013%m 18025449. 18025449 10.7326/0003-4819-147-10-200711200-00013

[11] Wales J. Last Song — Sharing Humanity while Maintaining Boundaries. N Engl J Med. 2019;381(20):1894–5. 10.1056/nejmp1907600. 31722149

[12] Dintino B, Raja A, Cafaro S. The race to save lives. The Boston Globe.

[13] Lamas DJ. I’m on the Front Lines. I Have No Plan for This. The New York Times.

[14] Martin N, MacQuarrie B, Pan D. Dispatches from the Front Lines. The Boston Globe.

[15] Miller D. Coronavirus anxiety overwhelmed this doctor. A deep breath helped. The Washington Post.

[16] Riordan K. From the front lines of the pandemic; The people too essential to isolate share how their lives have changed since the coronavirus hit — and what scares them about their jobs now. The Philadelphia Inquirer.

[17] The National Academy of Medicine. Taking Action Against Clinician Burnout: A Systems Approach to Professional Well-Being. National Academies; 2019. 31940160

[18] American Nurses Association. A Call to Action: Exploring Moral Resilience Toward a Culture of Ethical Practice. 2017.

[19] Farquhar MC, Ewing G, Booth S. Using mixed methods to develop and evaluate complex interventions in palliative care research. Palliat Med. 2011;25(8):748–57. 10.1177/0269216311417919. 21807749

[20] Creswell JW, Clark VLP. Designing and Conducting Mixed Methods Research. 3rd Edition ed. SAGE Publications, Inc; 2018.

[21] Denzin NK. Triangulation 2.0. J Mixed Methods Res. 2012;6(2):80–8. 10.1177/1558689812437186.

[22] Tashakkori A, Teddlie C, editors. SAGE Handbook of Mixed Methods in Social & Behavioral Research. 2nd Edition ed. SAGE Publications, Inc; 2010.

[23] Mohan D, Sacks OA, O'Malley J, A New Standard for Advance Care Planning (ACP) Conversations in the Hospital: Results from a Delphi Panel. J Gen Intern Med. Jan 2021;36(1):69–76. 10.1007/s11606-020-06150-0. 32816240 PMC7859119

[24] Courtright KR, Chivers C, Becker M, Electronic Health Record Mortality Prediction Model for Targeted Palliative Care Among Hospitalized Medical Patients: a Pilot Quasi-experimental Study. J Gen Intern Med. 2019;34(9):1841–7. 10.1007/s11606-019-05169-2. 31313110 PMC6712114

[25] Draucker CB, Martsolf DS, Poole C. Developing distress protocols for research on sensitive topics. Archives Psychiatric Nursing Oct. 2009;23(5):343–50. 10.1016/j.apnu.2008.10.008. 19766925

[26] Whitney C, Evered JA. The Qualitative Research Distress Protocol: A Participant-Centered Tool for Navigating Distress During Data Collection. Int J Qualitative Methods. 2022;2022/04/01:21:16094069221110317. 10.1177/16094069221110317.

[27] Bai X, Wang A, Cross W, Validation of the distress thermometer for caregivers of children and adolescents with schizophrenia. J Adv Nurs. 2020;76(2):687–98. 10.1111/jan.14233. 31600000

[28] Donovan KA, Grassi L, McGinty HL, Jacobsen PB. Validation of the Distress Thermometer worldwide: state of the science. Psycho-oncology. 2014;23(3):241–50. 10.1002/pon.3430. 25160838

[29] Haverman L, Van Oers HA, Limperg PF, Development and Validation of the Distress Thermometer for Parents of a Chronically Ill Child. J Pediatr. 2013;163(4):1140–e11462. 10.1016/j.jpeds.2013.06.011. 23910979

[30] Jacobsen PB, Donovan KA, Trask PC, Screening for psychologic distress in ambulatory cancer patients. Cancer Apr. 2005;1(7):1494–502. 10.1002/cncr.20940. 15726544

[31] Ma X, Zhang J, Zhong W, The diagnostic role of a short screening tool—the distress thermometer: a meta-analysis. Support Care Cancer. 2014;22(7):1741–55. 10.1007/s00520-014-2143-1. 24510195

[32] Thekkumpurath P, Venkateswaran C, Kumar M, Newsham A, Bennett MI. Screening for Psychological Distress in Palliative Care: Performance of Touch Screen Questionnaires Compared with Semistructured Psychiatric Interview. J Pain Symptom Manag. 2009;38(4):597–605. 10.1016/j.jpainsymman.2009.01.004. 19692204

[33] Wocial LD, Weaver MT. Development and psychometric testing of a new tool for detecting moral distress: the Moral Distress Thermometer. J Adv Nurs Jan. 2013;69(1):167–74. 10.1111/j.1365-2648.2012.06036.x. 22607094

[34] Gallup. Gallup 2019 Global Emotions Report. https://www.gallup.com/analytics/248906/gallup-global-emotions-report-2019.aspx

[35] NVivo qualitative data analysis software, (Version 14.23.4). Lumivero, 2024.

[36] Elo S, Kääriäinen M, Kanste O, Pölkki T, Utriainen K, Kyngäs H. Qualitative Content Analysis: A Focus on Trustworthiness. SAGE Open. 2014;4(1). 10.1177/2158244014522633.

[37] Korstjens I, Moser A, Series. Practical guidance to qualitative research. Part 2: Context, research questions and designs. Eur J Gen Pract. 2017;23(1):274–9. 10.1080/13814788.2017.1375090. 29185826 PMC8816399

[38] Sandelowski M. Whatever happened to qualitative description? Res Nurs Health. 2000;23(4):334–40. 10.1002/1098-240x(200008)23:4<334::aid-nur9>3.0.co;2-g. 10940958

[39] Graneheim UH, Lundman B. Qualitative content analysis in nursing research: concepts, procedures and measures to achieve trustworthiness. Nurse Educ Today Feb. 2004;24(2):105–12. 10.1016/j.nedt.2003.10.001. 14769454

[40] Hsieh HF, Shannon SE. Three approaches to qualitative content analysis. Qualitative Health Research Nov. 2005;15(9):1277–88. 10.1177/1049732305276687. 16204405

[41] Colorafi KJ, Evans B. Qualitative Descriptive Methods in Health Science Research. Health Environ Res Des J. 2016;9(4):16–25. 10.1177/1937586715614171. PMC7586301 26791375

[42] Korstjens I, Moser A, Series. Practical guidance to qualitative research. Part 4: Trustworthiness and publishing. Eur J Gen Pract. 2018;24(1):120–4. 10.1080/13814788.2017.1375092. 29202616 PMC8816392

[43] Genolini C, Ecochard R, Benghezal M, Driss T, Andrieu S, Subtil F. kmlShape: An Efficient Method to Cluster Longitudinal Data (Time-Series) According to Their Shapes. PLoS ONE. 2016;11(6):e0150738. 10.1371/journal.pone.0150738. 27258355 PMC4892497

[44] Genolini C, Falissard B. KmL: a package to cluster longitudinal data. Comput Methods Programs Biomed Dec. 2011;104(3):e112–21. 10.1016/j.cmpb.2011.05.008. 21708413

[45] Fetters MD, Curry LA, Creswell JW. Achieving Integration in Mixed Methods Designs-Principles and Practices. Health Serv Res. 2013;48(6pt2):2134–56. 10.1111/1475-6773.12117. 24279835 PMC4097839

[46] Younas A, Pedersen M, Durante A. Characteristics of joint displays illustrating data integration in mixed-methods nursing studies. J Adv Nurs Feb. 2020;76(2):676–86. 10.1111/jan.14264. 31713252

[47] Creswell JW, Fetters MD, Ivankova NV. Designing a mixed methods study in primary care. Ann Fam Med Jan-Feb. 2004;2(1):7–12. 10.1370/afm.104. 15053277 PMC1466635

[48] Guetterman TC, Fetters MD, Creswell JW. Integrating Quantitative and Qualitative Results in Health Science Mixed Methods Research Through Joint Displays. Annals Family Med. 2015;13(6):554–61. 10.1370/afm.1865. PMC4639381 26553895

[49] Allen R, Judkins-Cohn T, Develasco R, Moral Distress Among Healthcare Professionals at a Health System. JONA’S Healthc Law Ethics Regul. 2013;15(3):111–8. 10.1097/nhl.0b013e3182a1bf33. 23963112

[50] Altaker KW, Howie-Esquivel J, Cataldo JK. Relationships Among Palliative Care, Ethical Climate, Empowerment, and Moral Distress in Intensive Care Unit Nurses. American J Crit Care Jul. 2018;27(4):295–302. 10.4037/ajcc2018252. 29961665

[51] Bender MA, Andrilla CHA, Sharma RK, Hurd C, Solvang N, Mae-Baldwin L. Moral Distress and Attitudes About Timing Related to Comfort Care for Hospitalized Patients: A Survey of Inpatient Providers and Nurses. Am J Hospice Palliat Medicine^®^. 2019;36(11):967–73. 10.1177/1049909119843136. 30966758

[52] Mobley MJ, Rady MY, Verheijde JL, Patel B, Larson JS. The relationship between moral distress and perception of futile care in the critical care unit. Intensive Crit Care Nurs. 2007;23(5):256–63. 10.1016/j.iccn.2007.03.011. 17681468

[53] Sirilla J, Thompson K, Yamokoski T, Risser MD, Chipps E. Moral Distress in Nurses Providing Direct Patient Care at an Academic Medical Center. Worldviews Evid Based Nurs Apr. 2017;14(2):128–35. 10.1111/wvn.12213. 28253430

[54] Whitehead PB, Herbertson RK, Hamric AB, Epstein EG, Fisher JM. Moral Distress Among Healthcare Professionals: Report of an Institution-Wide Survey. J Nurs Scholarsh. 2015;47(2):117–25. 10.1111/jnu.12115. 25440758

[55] Forrest CB, Xu H, Thomas LE, Impact of the Early Phase of the COVID-19 Pandemic on US Healthcare Workers: Results from the HERO Registry. J Gen Intern Med. 2021. 10.1007/s11606-020-06529-z. PMC7946335 33694071

[56] Pappa S, Ntella V, Giannakas T, Giannakoulis VG, Papoutsi E, Katsaounou P. Prevalence of depression, anxiety, and insomnia among healthcare workers during the COVID-19 pandemic: A systematic review and meta-analysis. Brain Behav Immun. 2020;88:901–7. 10.1016/j.bbi.2020.05.026. 32437915 PMC7206431

[57] Maunder RG, Lancee WJ, Balderson KE, Long-term psychological and occupational effects of providing hospital healthcare during SARS outbreak. Emerg Infect Dis Dec. 2006;12(12):1924–32. 10.3201/eid1212.060584. 17326946 PMC3291360

[58] Shaukat N, Ali DM, Razzak J. Physical and mental health impacts of COVID-19 on healthcare workers: a scoping review. Int J Emerg Med. 2020;13(1). 10.1186/s12245-020-00299-5. PMC7370263 32689925

[59] Vindegaard N, Benros ME. COVID-19 pandemic and mental health consequences: Systematic review of the current evidence. Brain Behav Immun. May 30. 2020; 10.1016/j.bbi.2020.05.048 PMC7260522 32485289

[60] Lai J, Ma S, Wang Y, Factors Associated With Mental Health Outcomes Among Health Care Workers Exposed to Coronavirus Disease 2019. JAMA Netw Open. 2020;3(3):e203976. 10.1001/jamanetworkopen.2020.3976. 32202646 PMC7090843

[61] Li G, Miao J, Wang H, Psychological impact on women health workers involved in COVID-19 outbreak in Wuhan: a cross-sectional study. J Neurol Neurosurg Psychiatry Aug. 2020;91(8):895–7. 10.1136/jnnp-2020-323134. 32366684

[62] Rossi R, Socci V, Pacitti F, Mental Health Outcomes Among Frontline and Second-Line Health Care Workers During the Coronavirus Disease 2019 (COVID-19) Pandemic in Italy. JAMA Netw Open. 2020;3(5):e2010185. 10.1001/jamanetworkopen.2020.10185. 32463467 PMC7256664

[63] Roberts T, Daniels J, Hulme W, Psychological distress and trauma in doctors providing frontline care during the COVID-19 pandemic in the United Kingdom and Ireland: a prospective longitudinal survey cohort study. BMJ Open. 2021;11(7):e049680. 10.1136/bmjopen-2021-049680. PMC8275363 34244282

[64] Campbell SM, Ulrich CM, Grady C. A Broader Understanding of Moral Distress. American J Bioethics Dec. 2016;16(12):2–9. 10.1080/15265161.2016.1239782. 27901442

[65] Patch K, Huang C, Hendriks S, Wasserman D, Parrish M, Grady C. It's Pretty Sad If You Get Used to It: A Qualitative Study of First Responder Experiences with Opioid Overdose Emergencies. Prehosp Emerg Care Jul 18 2023:1–8. 10.1080/10903127.2023.2236200 PMC10794549 37436072

[66] Rushton CH, Kaszniak AW, Halifax JS. A framework for understanding moral distress among palliative care clinicians. J Palliat Med Sep. 2013;16(9):1074–9. 10.1089/jpm.2012.0490. 23777328

[67] McFarland DC. Less direct patient care delivered by medical trainees by the end of a hematology-oncology ward rotation: Association with empathy and related factors. Article. Psycho-oncology. 2019;28(6):1342–8. 10.1002/pon.5089. 30970150

[68] McFarland DC, Malone AK, Roth A. Acute empathy decline among resident physician trainees on a hematology–oncology ward: an exploratory analysis of house staff empathy, distress, and patient death exposure. Article. Psycho-oncology. 2017;26(5):698–703. 10.1002/pon.4069. 26776456

[69] Strote J, Schroeder E, Lemos J, Paganelli R, Solberg J, Range Hutson H. Academic Emergency Physicians' Experiences With Patient Death. Acad Emerg Med. 2011;18(3):255–60. 10.1111/j.1553-2712.2011.01004.x. 21401787

[70] Holland JM, Neimeyer RA. Reducing the risk of burnout in end-of-life care settings: The role of daily spiritual experiences and training. Palliat Supportive Care. 2005;3(3):173–81. 10.1017/s1478951505050297. 16594456

[71] White DM, Meeker MA. Guiding the Process of Dying: The Personal Impact on Nurses. J Hospice Palliat Nurs. 2019;21(5):390–6. 10.1097/NJH.0000000000000539. 30920491

[72] Lake ET, Narva AM, Holland S, Hospital nurses' moral distress and mental health during COVID-19. J Adv Nurs Mar. 2022;78(3):799–809. 10.1111/jan.15013. 34402538 PMC8447301

[73] Muir KJ, Porat-Dahlerbruch J, Nikpour J, Leep-Lazar K, Lasater KB. Top Factors in Nurses Ending Health Care Employment Between 2018 and 2021. JAMA Netw Open Apr. 2024;1(4):e244121. 10.1001/jamanetworkopen.2024.4121. PMC11004833 38592723

[74] Chooljian DM, Hallenbeck J, Ezeji-Okoye SC, Sebesta R, Iqbal H, Kuschner WG. Emotional Support for Health Care Professionals: A Therapeutic Role for the Hospital Ethics Committee. J Soc Work End-of-Life Palliat Care. 2016;12(3):277–88. 10.1080/15524256.2016.1200519. 27462956

[75] Davidson JE, Graham P, Montross-Thomas L, Norcross W, Zerbi G. Code Lavender: Cultivating Intentional Acts of Kindness in Response to Stressful Work Situations. EXPLORE. 2017;13(3):181–5. 10.1016/j.explore.2017.02.005. 28668136

[76] Stone RSB, Code Lavender. Nursing. 2018;48(4):15–7. 10.1097/01.nurse.0000531022.93707.08. 29561364

[77] Hamric AB, Epstein EG. A Health System-wide Moral Distress Consultation Service: Development and Evaluation. HealthCare Ethics Comm Forum Jun. 2017;29(2):127–43. 10.1007/s10730-016-9315-y. 28070806

[78] Edrees H, Connors C, Paine L, Norvell M, Taylor H, Wu AW. Implementing the RISE second victim support programme at the Johns Hopkins Hospital: a case study. BMJ Open Sep. 2016;30(9):e011708. 10.1136/bmjopen-2016-011708. PMC5051469 27694486

[79] Browning ED, Cruz JS. Reflective Debriefing: A Social Work Intervention Addressing Moral Distress among ICU Nurses. J Soc Work End-of-Life Palliat Care. 2018;14(1):44–72. 10.1080/15524256.2018.1437588. 29488856

[80] Edmonds KP, Yeung HN, Onderdonk C, Mitchell W, Thornberry K. Clinical Supervision in the Palliative Care Team Setting: A Concrete Approach to Team Wellness. J Palliat Med. 2015;18(3):274–7. 10.1089/jpm.2014.0248. 25517027

[81] Jonas DF, Bogetz JF. Identifying the Deliberate Prevention and Intervention Strategies of Pediatric Palliative Care Teams Supporting Providers during Times of Staff Distress. J Palliat Med. 2016;19(6):679–83. 10.1089/jpm.2015.0425. 27167894

[82] Orellana-Rios CL, Radbruch L, Kern M, Mindfulness and compassion-oriented practices at work reduce distress and enhance self-care of palliative care teams: a mixed-method evaluation of an on the job program. BMC Palliat Care. 2018;17(1). 10.1186/s12904-017-0219-7. PMC5501358 28683799

[83] Vahey DC, Aiken LH, Sloane DM, Clarke SP, Vargas D. Nurse Burnout and Patient Satisfaction. Med Care. 2004;42(2):II–57. 10.1097/01.mlr.0000109126.50398.5a. PMC2904602 14734943

[84] Forbes D, Lewis V, Varker T, Psychological First Aid Following Trauma: Implementation and Evaluation Framework for High-Risk Organizations. Psychiatry: Interpers Biol Processes. 2011;74(3):224–39. 10.1521/psyc.2011.74.3.224. 21916629

[85] Thum CC, Chai YC, Zaman Huri S, Wan Nawawi WZ, Ibrahim N. Innovative psychological first aid (PFA) in the new normal for frontliners. Perspect Psychiatr Care. 2020. 10.1111/ppc.12600. 32770539

[86] Shah K, Bedi S, Onyeaka H, Singh R, Chaudhari G. The Role of Psychological First Aid to Support Public Mental Health in the COVID-19 Pandemic. Cureus. 2020. 10.7759/cureus.8821. PMC7384717 32742836

[87] Sulaiman AH, Ahmad Sabki Z, Jaafa MJ, Development of a Remote Psychological First Aid Protocol for Healthcare Workers Following the COVID-19 Pandemic in a University Teaching Hospital, Malaysia. Healthcare. 2020;8(3):228. 10.3390/healthcare8030228. 32722042 PMC7551586

[88] Rosa WE, Roberts KE, Schlak AE, The Critical Need for a Meaning-Centered Team-Level Intervention to Address Healthcare Provider Distress Now. Int J Environ Res Public Health Jun. 2022;25(13). 10.3390/ijerph19137801. PMC9265276 35805459

[89] Fillion L, Duval S, Dumont S, Impact of a meaning-centered intervention on job satisfaction and on quality of life among palliative care nurses. Psycho-Oncology: J Psychol Social Behav Dimensions Cancer. 2009;18(12):1300–10. 10.1002/pon.1513 19165757

[90] Snyder DJ, Mournet AM, Pao M. Reflections on experiential training in meaning-centered psychotherapy: How MCP ended up facilitating professional wellbeing. Palliat Support Care Feb. 2023;21(1):38–42. 10.1017/S1478951522000414. 35451355 PMC11285004

[91] Fuehrer S, Weil A, Osterberg LG, Zulman DM, Meunier MR, Schwartz R. Building Authentic Connection in the Patient-Physician Relationship. J Prim Care Community Health. 2024;15 doi. 10.1177/21501319231225996. PMC10823846 38281122

[92] Kowalski C, Nitzsche A, Scheibler F, Steffen P, Albert U-S, Pfaff H. Breast cancer patients’ trust in physicians: The impact of patients’ perception of physicians’ communication behaviors and hospital organizational climate. Patient Educ Couns. 2009;77(3):344–8. 10.1016/j.pec.2009.09.003. 19818577

[93] Dyrbye LN, Burke SE, Hardeman RR, Association of Clinical Specialty With Symptoms of Burnout and Career Choice Regret Among US Resident Physicians. JAMA. 2018;320(11):1114–30. 10.1001/jama.2018.12615. 30422299 PMC6233627

[94] Maslach C, Leiter MP. Understanding the burnout experience: recent research and its implications for psychiatry. World Psychiatry. 2016;15(2):103–11. 10.1002/wps.20311. 27265691 PMC4911781

[95] Strout TD, Hillen M, Gutheil C, Tolerance of uncertainty: A systematic review of health and healthcare-related outcomes. Patient Educ Couns. 2018;101(9):1518–37. 29655876 10.1016/j.pec.2018.03.030

[96] Hillen MA, Gutheil CM, Strout TD, Smets EMA, Han PKJ. Tolerance of uncertainty: Conceptual analysis, integrative model, and implications for healthcare. Soc Sci Med. 2017;180:62–75. 10.1016/j.socscimed.2017.03.024. 2017/05/01/. 28324792

[97] Aiken LH, Clarke SP, Sloane DM, Sochalski J, Silber JH. Hospital Nurse Staffing and Patient Mortality, Nurse Burnout, and Job Dissatisfaction. JAMA. 2002;288(16):1987–93. 10.1001/jama.288.16.1987. 12387650

[98] Aiken LH, Lasater KB, Sloane DM, Physician and Nurse Well-Being and Preferred Interventions to Address Burnout in Hospital Practice. JAMA Health Forum. 2023;4(7):e231809. 10.1001/jamahealthforum.2023.1809. 37418269 PMC10329209

[99] Lasater KB, Aiken LH, Sloane DM, Chronic hospital nurse understaffing meets COVID-19: an observational study. BMJ Qual Saf. 2021;30(8):639–47. 10.1136/bmjqs-2020-011512. PMC7443196 32817399

